# Adaptive scan duration in SPECT: Evaluation for radioembolization

**DOI:** 10.1002/mp.14095

**Published:** 2020-03-10

**Authors:** Martijn M. A. Dietze, Britt Kunnen, Casper Beijst, Hugo W. A. M. de Jong

**Affiliations:** ^1^ Radiology and Nuclear Medicine Utrecht University and University Medical Center Utrecht Utrecht the Netherlands; ^2^ Image Sciences Institute Utrecht University and University Medical Center Utrecht Utrecht the Netherlands

**Keywords:** adaptive scan, patient‐specific scan duration, SPECT

## Abstract

**Purpose:**

It may be challenging to select the optimal scan duration for single‐photon emission computed tomography (SPECT) protocols because the activity distribution characteristics can differ in every scan. Using simulations and experiments, we investigated whether the scan duration can be optimized for every scan separately by evaluating the activity distribution during scanning. We refer to this as adaptive scanning.

**Methods:**

The feasibility of adaptive scanning was evaluated for the detection of extrahepatic depositions in the pretreatment procedure of radioembolization, in which ^99m^Tc‐labeled macroaggregated albumin (^99m^Tc‐MAA) is injected into the liver. We simulated fast 1‐min detector rotations and updated the reconstruction with the newly collected counts after every rotation. The scan was terminated when one of the two criteria was met: (a) when the mask difference of the detected extrahepatic deposition between two consecutive rotations was lower than 5%; or (b) when the reconstructed extrahepatic activity was negligible with respect to the total reconstructed activity (<0.075%). The performance of adaptive scanning was evaluated using a digital phantom with various activity distributions, a physical phantom experiment, and simulations based on 129 patient activity distributions.

**Results:**

The digital phantom data showed that the scan termination times substantially depended on the activity distribution characteristics. The experimental phantom data showed the feasibility of adaptive scanning with physical scanner measurements and illustrated that fast detector motion was not limiting the adaptive scanning performance. The patient data showed a large spread in the scan terminations times. By adaptive scanning, the mean scan duration of the patient distributions was shortened from 20 min (current clinical protocol) to 4.8 ± 0.2 min. The detection accuracy of extrahepatic depositions was unaffected and the mean difference in the extrahepatic deposition masks (compared with the 20‐min scan) was only 7.0 ± 1.0%.

**Conclusion:**

Our study suggests that the SPECT scan duration can be personalized by assessing the activity distribution characteristics during scanning for the detection of extrahepatic depositions in the pretreatment procedure of radioembolization. The adaptive scanning approach might also be of benefit for other SPECT protocols, as long as a measure of interest is available for optimization.

## Graphical abstract

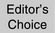

## 1 Introduction

Selecting the optimal scan duration for a single‐photon emission computed tomography (SPECT) imaging task remains a challenge because the activity distribution characteristics can differ in every scan. To ensure that the reconstruction answers the imaging question in all cases (i.e., also with low activity or heterogeneous small volumes), scanning is performed with a fixed acquisition time that may be longer than required for most distributions. The scanning efficiency and workflow can be improved if the scan duration is optimized for every activity distribution separately, that is, by scanning shorter for simple activity distributions and longer for complicated ones. We refer to this as adaptive scanning.

We aim to evaluate an adaptive scanning technique by performing multiple fast detector rotations (e.g., 1 min each) so that the reconstruction can be updated with the extra obtained counts after every rotation. Central to this approach is the existence of a metric of image quality for the task at hand that can be automatically measured. The change in this metric is tracked over the rotations and when its relative change falls below a certain threshold value, the scan is considered to be converged (with respect to the imaging task) and can be terminated. This approach ensures that all activity distributions satisfy the imaging requirement but also that the scan duration is not longer than necessary.

This study evaluates the possibility for such an adaptive scan duration for the pretreatment procedure of hepatic radioembolization, in which ^99m^Tc‐labeled macroaggregated albumin (^99m^Tc‐MAA) is injected into the liver to estimate the activity distribution of the ^90^Y microspheres.^1^ The pretreatment procedure has two objectives in current clinical practice: (a) to determine the fraction of activity shunting to the lungs and (b) to detect potential extrahepatic depositions.

Previously, it was demonstrated that the lung shunt fraction (LSF) can be accurately determined within minutes of scanning (since it is based on the total counts in relatively large volumes)^2^ and no further optimization is hence required for this task. However, for the detection of extrahepatic depositions, count statistics become more important. These count statistics are highly dependent on the specific activity distribution characteristics (e.g., the activity in an extrahepatic deposition and the background noise), which can vary considerably between patients. Hence, it is expected that a substantial spread in the required scan durations will be observed for this imaging task.

This study will investigate the influence of the activity distribution characteristics on the scan termination times with a simulation study and the performance in clinically encountered distributions with a retrospective patient study. The fast scanning protocol requires continuous detector motion over the multiple rotations in order to reduce the overhead time when compared with regular step‐and‐shoot motion. Since the detector orbit can fluctuate over the rotations and the detector distance and detector rotation angle can fluctuate within one projection, image artifacts or degrading effects may occur. The magnitude of these effects will be evaluated in a phantom study.

## 2 Methods

### 2.1 Extrahepatic deposition detection

To distinguish an extrahepatic deposition from a noisy background, a more or less arbitrary elevation of the reconstruction voxel activity and shape is normally assessed by the physician. Our proposed method is an automatic detection approach which we base on work originating from our institute with the same acquisition and reconstruction parameters as in this study.^3^

**Fig. 1:**
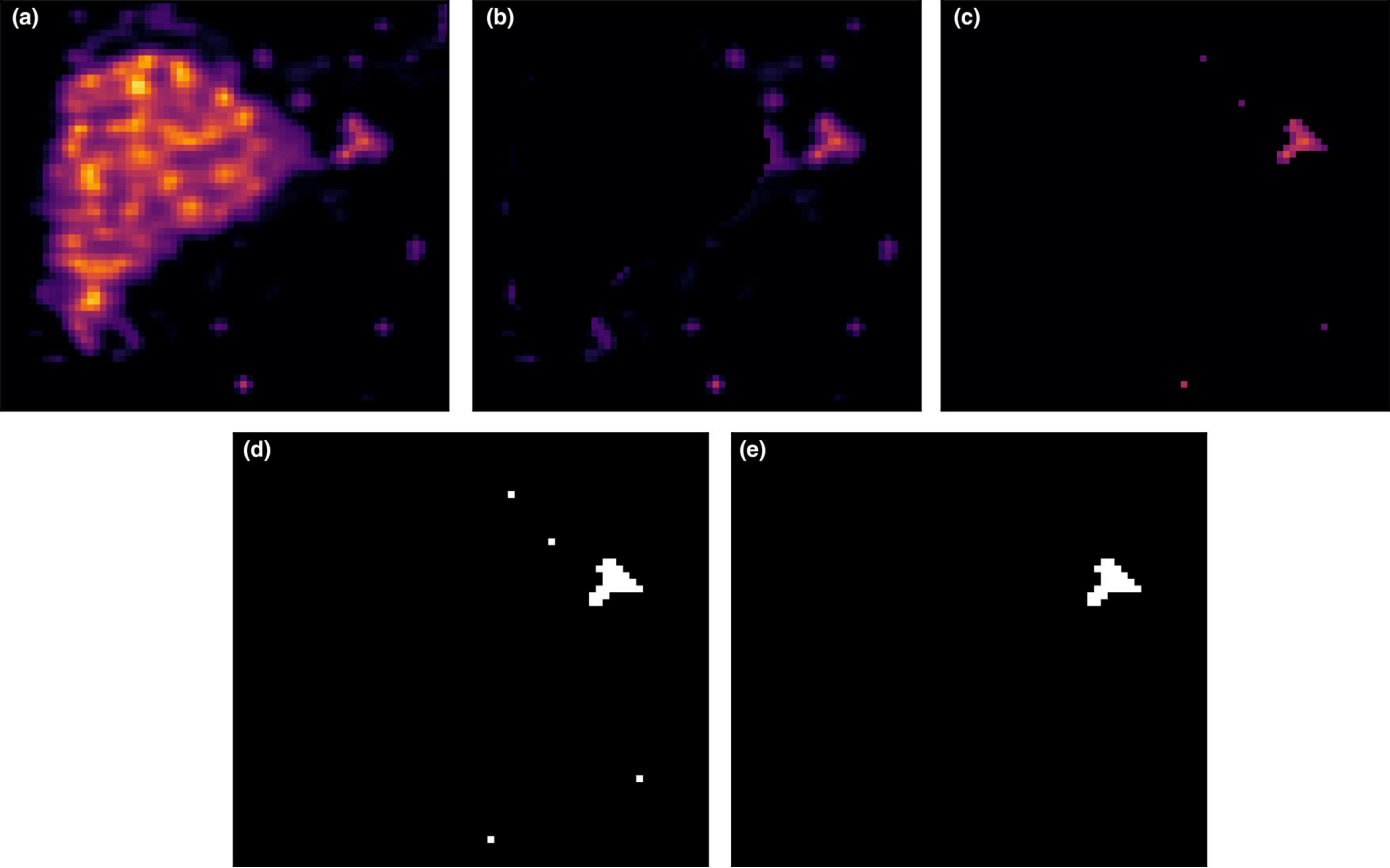
An illustration of the automatic process for detecting potential extrahepatic depositions. (a) The initial reconstructed image. (b) The lungs and liver are removed from the reconstruction. (c) The concentration threshold is applied. (d) The remaining voxels with activity are merged into clusters. (e) The threshold for the total activity present in a deposition is applied. [Color figure can be viewed at wileyonlinelibrary.com]

The process of detecting potential extrahepatic depositions is illustrated in Fig. 1. From the initially reconstructed image [Fig. 1(a)], only the background is selected by removing the lung and liver masks (dilated by 2 cm) from the image [Fig. 1(b)]. These masks are expected to be known in advance. A threshold on the activity concentration (the voxel concentration must be greater than 0.017% of the total injected activity per ml, as is the lowest concentration observed in an extrahepatic deposition^3^) is hence applied [Fig. 1(c)]. The remaining voxels with activity are merged into clusters [Fig. 1(d)]. The total activity in every cluster is hence measured and the clusters which have an activity greater than 0.1% of the total reconstructed activity (the lowest activity observed in an extrahepatic deposition^3^) are considered to be an extrahepatic deposition [Fig. 1(e)]. The entire process is performed in 3D.

### 2.2 Scan duration algorithm

The adaptive scan protocol is performed by performing fast detector rotations of 1 min each. The reconstruction is updated with the newly collected counts after every rotation. The following two criteria are evaluated for every intermediate reconstruction and the scan is terminated when one of them is met:

1. The total reconstructed activity in the background mask is lower than 0.075% of the total reconstructed activity.

The rationale behind the first criterion is that there cannot be an extrahepatic deposition if the activity in the background is negligible with respect to the total activity. The threshold of 0.075% was chosen because this is a defined summed extrahepatic activity threshold (0.1%) minus a margin to account for noise fluctuations.

- The relative difference in the mask of a detected extrahepatic deposition between two subsequent rotations (measured by calculating the total number of changed voxels between the two masks (i.e., from being part of the mask to being left out; or vice versa) and divided by the total number of voxels in the most recent mask) is lower than 5%.

The rationale behind the second criterion is that the reconstruction will change substantially in the first few rotations because noise has a major impact. As the rotations progress, however, the reconstruction will become increasingly stable and hence the difference between two rotations will become increasingly smaller. The rate of this convergence is expected to depend on the activity distribution characteristics and will differ in every scan. We believe that, in clinical practice, it is most important to be confident in the shape and location of an extrahepatic deposition so that its origin can be accurately determined. Hence, we will track the mask of the detected extrahepatic deposition over time to serve as a surrogate measure for these properties. The threshold of this extrahepatic mask change is a trade‐off between accuracy and termination speed; we used 5% because with this setting all extrahepatic depositions were accurately detected in the patient study.

### 2.3 Simulation study

A simulation study was performed to study the activity distribution characteristics that influence the scan termination times. The XCAT phantom program^4^ was used to simulate a patient distribution. The liver and lungs were uniformly filled with the LSF set to 5.0% (such fractions are often encountered^5^). A spherical extrahepatic deposition was positioned close to the stomach (a common location for extrahepatic depositions^3^). The total activity in the phantom was fixed at 150 MBq ^99m^Tc (the currently injected activity at our institute^1^).

We varied the extrahepatic deposition’s activity concentration, volume, and location (by moving the extrahepatic deposition downward over various distances from the reference location). Only one parameter was changed at a time (see Table I for all studied options). The reference phantom had an extrahepatic deposition activity concentration of 0.10 MBq/ml, an extrahepatic deposition volume of 15.19 ml, and its extrahepatic deposition positioned at the reference location. Such phantom configurations are representative of clinical cases.^3^

**Table 1:** The studied configurations of the XCAT phantom in which one parameter was changed at a time in relation to the reference phantom. The reference phantom was created to be the representative of clinical cases.^3^

	Extrahepatic concentration (Liver concentration) (MBq/ml)	Extrahepatic volume (ml)	Extrahepatic location shift (cm)
Reference	0.10 (0.08)	15.19	0.0
Concentration changes	0.05 (0.08)	15.19	0.0
	0.20 (0.08)	15.19	0.0
	0.50 (0.07)	15.19	0.0
Volume changes	0.10 (0.08)	4.79	0.0
	0.10 (0.08)	7.27	0.0
	0.10 (0.08)	30.44	0.0
Location shifts	0.10 (0.08)	15.19	1.95
	0.10 (0.08)	15.19	3.90
	0.10 (0.08)	15.19	7.79

The phantom was configured on a 128 × 128 × 128 grid with a 3.9 mm isotropic voxel size. The nuclear projections were simulated with the Utrecht Monte Carlo System (UMCS),^6^, ^7^ which includes realistic physics for photon interactions in the patient and detector. The photopeak window was set to 129–150 keV and the scatter window to 108–129 keV. The projector simulated a dual‐head scanner with a step‐and‐shoot body‐tracing orbit (with a 1 cm patient‐detector gap), a low‐energy high‐resolution (LEHR) collimator, and 120 acquisition angles over 360°.

Rotations of 1 min each were simulated by the addition of Poisson noise (scaled to the total phantom activity) and background noise (obtained from a scan with no activity present) to the projections. For every phantom configuration, 20 rotations were evaluated by consecutively adding new counts to the projections.

The reconstructions were made with UMCS using the OSEM reconstruction algorithm with six iterations and eight subsets, dual‐energy window scatter correction (*k* = 0.5), and a post‐reconstruction Gaussian filter of 5 mm full width at half maximum (FWHM). Attenuation correction was performed with the μ‐map obtained from the low‐dose CT. These reconstruction parameters are the current clinical protocol in our institute.

The scan duration algorithm was then used to determine the optimal scan termination times for every phantom configuration. The mask and activity of the detected extrahepatic deposition were determined in the reconstruction at termination time and the reconstruction at 20 min, and their differences were calculated. For every phantom configuration, ten noise realizations were performed to study the stability of the results.

### 2.4 Phantom study

**Fig. 2:**
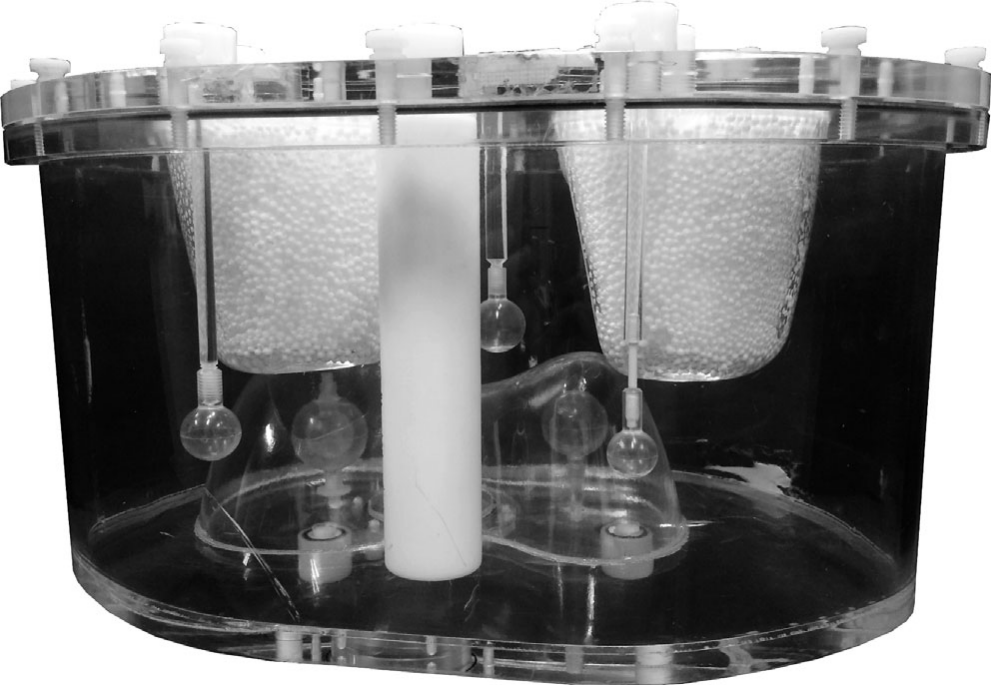
The anthropomorphic phantom with liver and lung compartments, and three extrahepatic depositions placed in the background compartment.

A phantom study was performed to evaluate the performance of adaptive scanning for physical scanner measurements and to study the influence of the fast continuous detector motion. An anthropomorphic phantom (IEL, Chilcompton, UK, Model ECT/TOR/P) was altered by the inclusion of three extrahepatic depositions (see Fig. 2). A hot sphere and hot sphere with a cold core were present in the liver compartment. The lungs were filled with an LSF of 5.2% and the total activity in the phantom was 154 MBq. The deposition‐to‐liver activity concentration ratio was 2.86. The individual activity levels are shown in Table II.

**Table 2:** The configuration of the phantom experiment. The background volume had no activity present.

	Concentration (MBq/ml)	Volume (ml)	Activity (MBq)
Liver	0.098	1172	115
Lungs	0.012	690	8
Hot sphere	0.78	15.9	12.4
Hot sphere with cold core	0.78	18.9	14.8
Extrahepatic deposition 1	0.28	2.0	0.56
Extrahepatic deposition 2	0.28	4.2	1.18
Extrahepatic deposition 3	0.28	8.2	2.30

Scanning was performed on a Siemens Symbia T16 system by performing 20 rotations of 1 min each in continuous motion. The acquisition and reconstruction parameters were (except for the fast continuous detector motion) the same as in the simulation study. The scan duration algorithm was used to determine the optimal scan termination times and the extrahepatic mask and activity differences with the 20‐min scan were measured.

Some resolution degradation is expected when scanning with fast continuous detector motion because the detector orbit can fluctuate over the rotations and the detector‐patient distance and the detector rotation angle can fluctuate within one bin. To evaluate the magnitude of the detector orbit fluctuations, the detector distance was measured over the 20 rotations and its standard deviation was calculated. To evaluate the influence of the continuous motion, the phantom was also scanned with a single‐rotation step‐and‐shoot 20‐min scan. The reconstruction of this scan was visually and quantitatively compared to the reconstruction of 20 one‐min rotations.

### 2.5 Patient study

The potential for adaptive scanning was then evaluated for clinical patient activity distributions. In total, 129 patient projections with corresponding attenuation maps and body, lung, and liver delineations were used. All patients received an injection of approximately 150 MBq ^99m^Tc‐MAA (0.8 mg, Technescan LyoMAA, Mallinckrodt Medical B.V., Petten, the Netherlands) in accordance with the radioembolization guidelines.^8^ The acquisition and reconstruction parameters were the same as in the simulation study.

Since the patient scans were performed with a single 20‐min rotation, 20 one‐min rotations were created by subsampling uniform random subsets from the 20‐min projection: all counts of the 20‐min projection were considered individual events and their positions (x, y, angle) were put in an event list‐mode. This list‐mode was shuffled randomly and the first (or second, etc.) 5% of this new list‐mode was used to create the 20 one‐min acquisitions. The sampling process was repeated ten times to study the stability of the reconstructions to different noise distributions.

The scan duration algorithm was used to determine the optimal scan termination time for every activity distribution. The extrahepatic deposition mask and activity differences with the 20‐min scan were measured. For every detected extrahepatic deposition, the contrast‐to‐noise ratio was calculated by dividing the contrast between the extrahepatic deposition mask and background mask by the standard deviation of the background mask. The background mask was available from the automatic extrahepatic deposition detection methodology and consisted of the body contour from which the liver, lung, and all detected extrahepatic deposition masks were subtracted. The analyses were performed for the ten noise realizations separately.

For comparison, the scan duration was also shortened without separate optimization for every patient activity distribution (i.e., by scanning with a single rotation with the same shortened scan duration for all patients). The scan duration was gradually decreased and it was determined until which the scan duration of all extrahepatic depositions was accurately detected. An extrahepatic deposition was considered to be accurately detected when the automatic detection algorithm retrieved the same number of extrahepatic depositions as in the 20‐min scan and for those masks, the difference in their center of mass was lower than 5 pixels.

## 3 Results

### 3.1 Simulation study

**Fig. 3:**
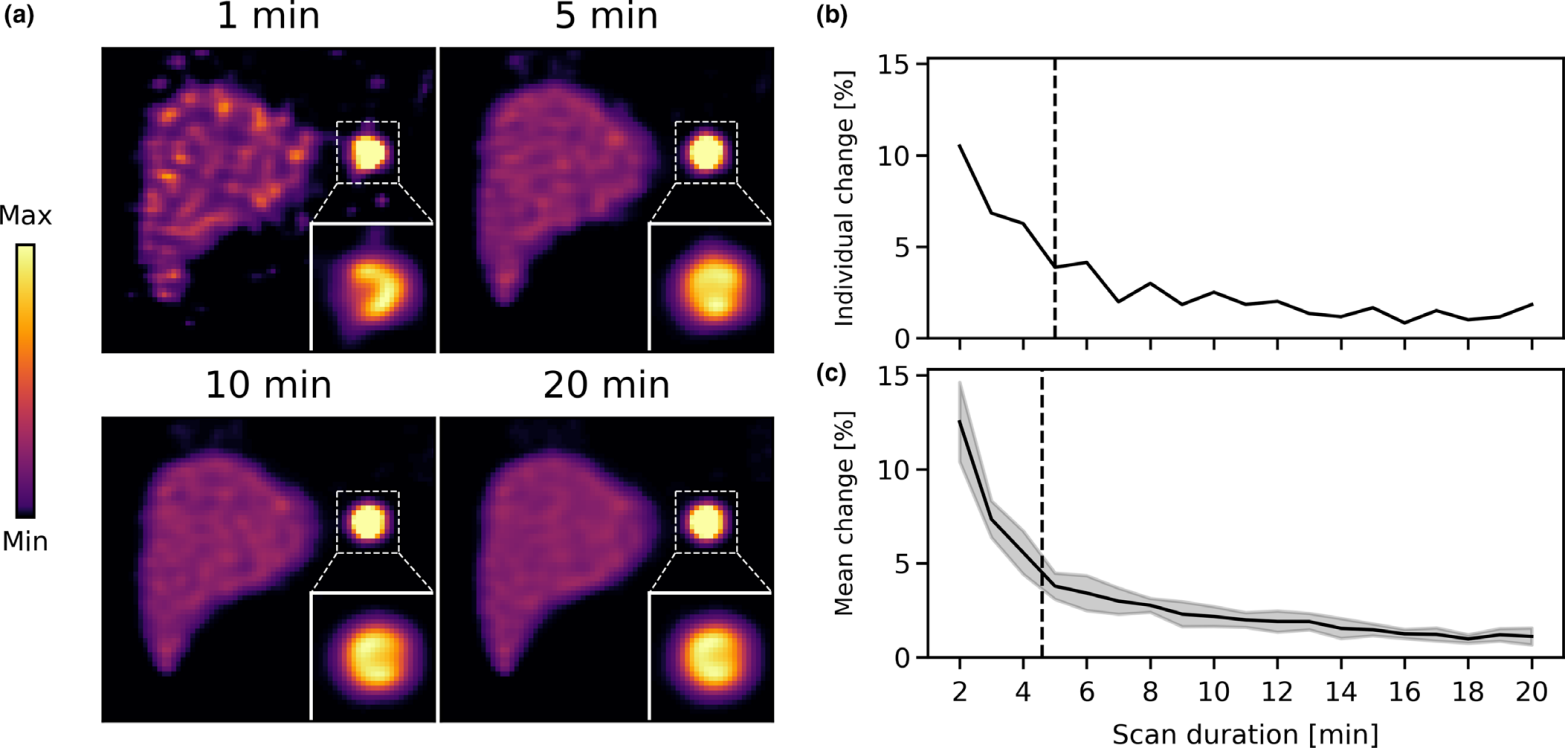
(a) Slices from the reconstructions for four scan durations for the XCAT phantom with 0.50 MBq/ml extrahepatic deposition activity concentration. The image maximum was set to 5x the liver concentration. (b) The relative change in the mask of the extrahepatic deposition, as a function of scan duration. The black dashed line indicates the scan termination time. (c) The mean relative change in the mask for ten (individually analyzed) noise realizations. The shaded error bars indicate the standard deviation. The black dashed line indicates the mean scan termination time. [Color figure can be viewed at wileyonlinelibrary.com]

Examples of the reconstructions obtained from the XCAT phantom with an extrahepatic deposition activity concentration of 0.50 MBq/ml are shown in Fig. 3(a). Some differences are observed in the extrahepatic deposition shape and size between the 1 and 5‐min scans. After 5 mins, however, almost no changes are observed.

The relative change in the mask of the extrahepatic deposition is shown in Fig. 3(b) [for the scan of Fig. 3(a)]. The relative change dropped below 5% for a scan duration of 5 min, which is in agreement with the visual observation from Fig. 3(a). The mean mask changes in the ten (individually analyzed) noise realizations are shown in Fig. 3(c). The mean scan termination time was 4.6 ± 0.5 min, which shows that the proposed algorithm provides (for this phantom configuration) relatively stable results.

**Fig. 4:**
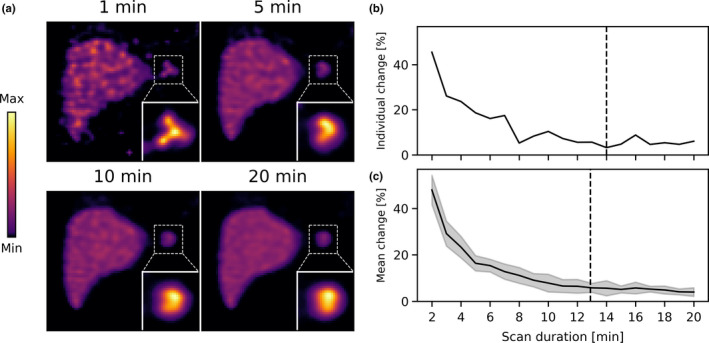
(a) Slices from the reconstructions for four scan durations for the XCAT phantom with 0.05 MBq/ml extrahepatic deposition activity concentration. The image maximum was set to 5x the liver concentration. (b) The relative change in the mask of the extrahepatic deposition, as a function of scan duration. The black dashed line indicates the scan termination time. (c) The mean relative change in the mask for ten (individually analyzed) noise realizations. The shaded error bars indicate the standard deviation. The black dashed line indicates the mean scan termination time. [Color figure can be viewed at wileyonlinelibrary.com]

The same evaluation as described above was performed on the phantom with 0.05 MBq/ml (instead of 0.50 MBq/ml as above). Slices from the obtained reconstructions are shown in Fig. 4(a). Visually, the extrahepatic deposition changed considerably more over the scan duration. This behavior is also found in the graphs on the relative change in the mask [Fig. 4(b) and 4(c)]: the phantom configuration reached the 5% threshold level after 12.9 ± 2.8 min, which is substantially longer than in the previous phantom configuration. This phantom comparison illustrates that the activity distribution characteristics have a substantial influence on the scan termination time.

The scan termination times and corresponding differences in the masks (compared with the 20‐min scan) are shown in Table III for all studied phantom configurations. The predominant factor for the scan termination time was the activity concentration of the extrahepatic deposition: depositions with a higher activity concentration terminated earlier. The volume of the extrahepatic deposition had some influence: larger extrahepatic depositions terminated earlier. The location of the extrahepatic deposition showed no substantial influence.

**Table 3:** The scan termination times of the studied extrahepatic deposition configurations. The difference in the extrahepatic deposition mask and the extrahepatic activity compares the scan with its duration from adaptive scanning with the 20‐min scan. The standard deviations were retrieved from the ten (individually analyzed) noise realizations.

Extrahepatic deposition configuration	Scan termination (min)	Mask difference (%)	Activity difference (%)
Concentration (MBq/mL)			
0.05	12.9 ± 2.8	11.9 ± 3.3	4.0 ± 1.6
0.10	8.8 ± 1.7	9.7 ± 2.1	2.0 ± 1.2
0.20	7.3 ± 0.9	9.3 ± 1.7	1.4 ± 1.4
0.50	4.6 ± 0.5	7.7 ± 1.0	1.5 ± 1.0
Volume (ml)			
4.79	9.3 ± 3.1	15.8 ± 6.8	4.3 ± 2.8
7.27	9.1 ± 1.6	9.6 ± 3.3	2.7 ± 1.8
15.19	8.8 ± 1.7	9.7 ± 2.1	2.0 ± 1.2
30.44	7.9 ± 0.9	8.5 ± 2.3	0.8 ± 0.7
Location shift (cm)			
0.00	8.8 ± 1.7	9.7 ± 2.1	2.0 ± 1.2
1.95	8.2 ± 1.7	9.0 ± 2.4	1.5 ± 1.1
3.90	6.7 ± 0.8	11.8 ± 2.7	3.0 ± 1.6
7.79	7.5 ± 1.4	10.3 ± 2.4	2.8 ± 1.8

### 3.2 Phantom study

**Fig. 5:**
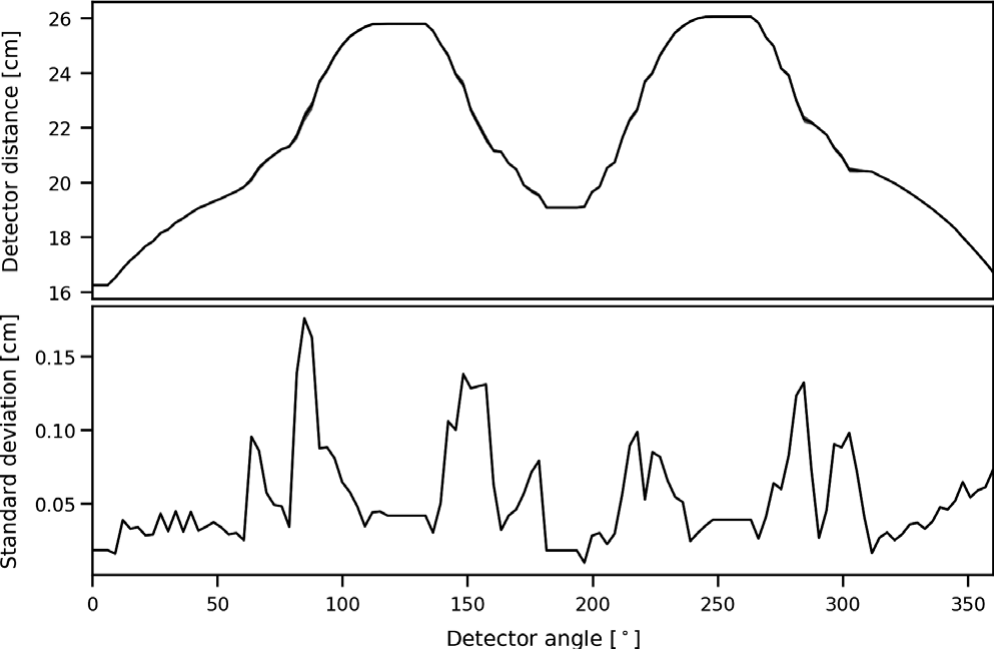
The mean and standard deviation of the detector distance as a function of the detector angle for the 20 one‐min scans in continuous motion.

The mean detector orbit of the 20 rotations in the phantom study is shown together with the corresponding standard deviation in Fig. 5. The standard deviation was small in relation to the detector distance mean, which indicates that the detector orbit was able to reproduce well over the multiple rotations.

**Fig. 6:**
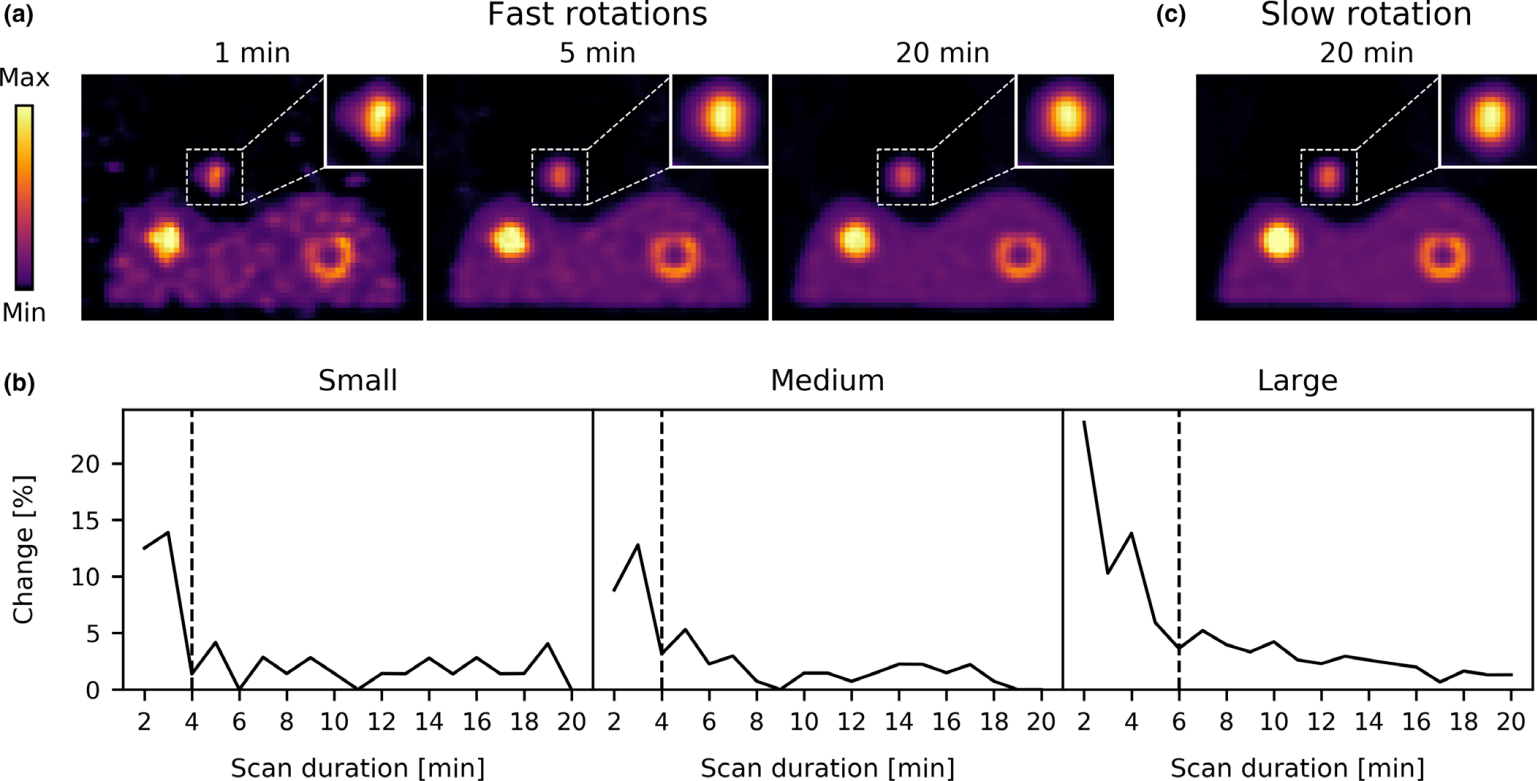
(a) Slices from the reconstructions (shown is the slice with the large extrahepatic deposition) of the phantom experiment obtained from three scan durations with fast rotations and (c) from a slow full‐duration scan. (b) The relative change in the extrahepatic deposition mask, as a function of scan duration. The black dashed lines indicate the scan durations in which a 5% change between two subsequent rotations was achieved. [Color figure can be viewed at wileyonlinelibrary.com]

Slices from the reconstructions of the phantom experiment with varying scan durations are shown in Fig. 6(a). There is a substantial change in the shape of the extrahepatic deposition between the scan of 1 and 5 min in duration. However, between 5 and 20 min of scan duration, hardly any differences can be observed.

This behavior is also found in the graphs on the relative change in the extrahepatic deposition mask in Fig. 6(b), which shows that the 5% threshold level was reached after 4, 4, and 6 min for the small, medium, and large extrahepatic depositions, respectively. After 6 min of scanning, the extrahepatic mask differences with the 20‐min scan were 8.1%, 4.4%, and 8.2% and the activity differences were 4.7%, 2.3%, and 1.2% for the small, medium, and large extrahepatic depositions, respectively. This indicates that, also for the phantom experiment, the scan might also have been earlier terminated.

The reconstruction of the single‐rotation 20‐min scan is shown in Fig. 6(c). It can be observed that the reconstruction from the single‐rotation is somewhat sharper than the one obtained with multiple fast rotations: the intrahepatic deposition is more uniform and the deposition with cold core is slightly more pronounced. The contrast‐to‐noise ratio (CNR) of the intrahepatic deposition was 38.4 for the single‐rotation 20‐min scan and 35.4 for the one obtained with multiple fast rotations. We conclude that fast detector motion gives rise to some resolution degradation, but will ultimately not be limiting the feasibility of adaptive scanning.

### 3.3 Patient study

**Fig. 7:**
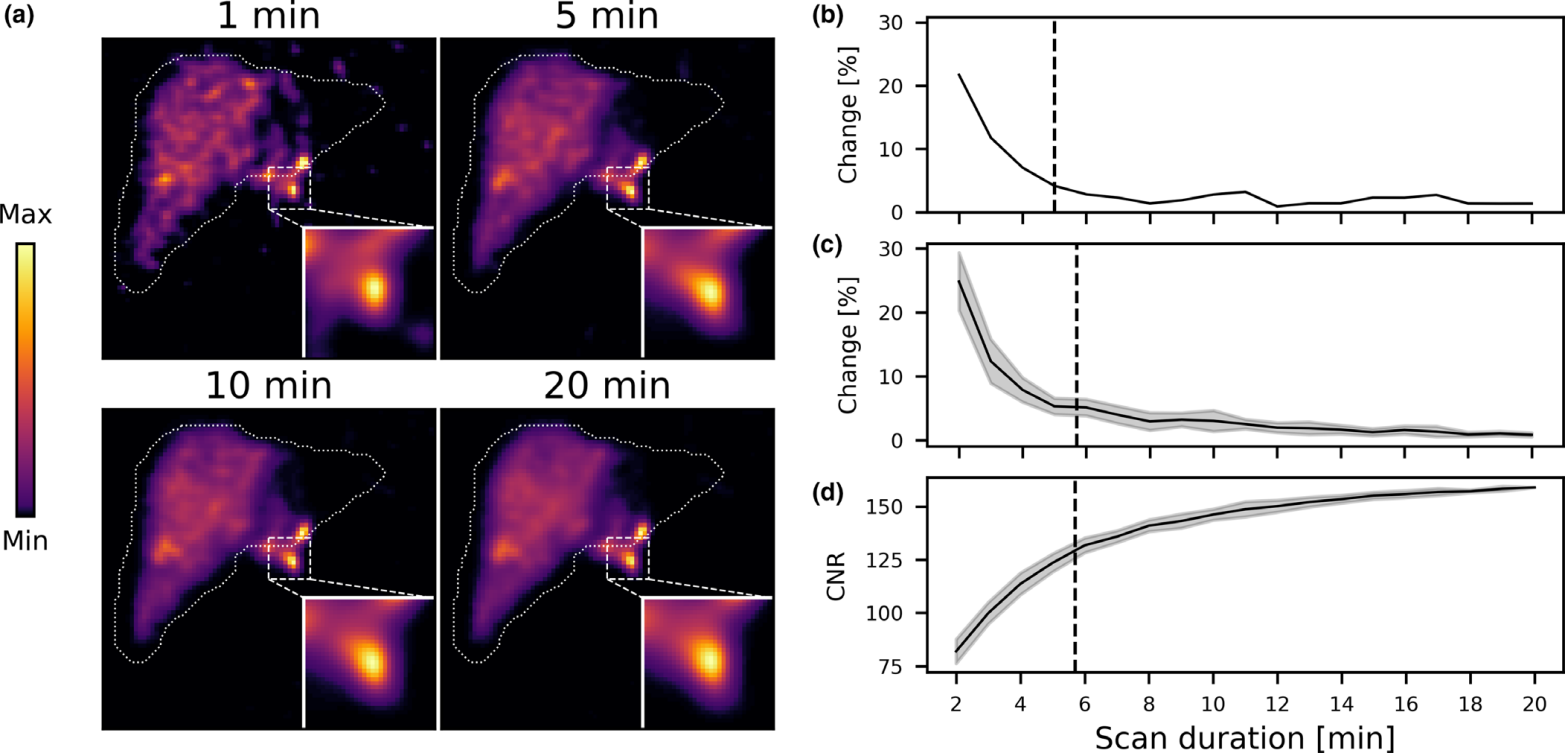
(a) Slices from the reconstructions for four scan durations for a patient activity distribution with a high‐concentration extrahepatic deposition. (b) The relative change in the mask of the extrahepatic deposition, as a function of scan duration. The black dashed line indicates the scan termination time. (c) The mean relative change in the extrahepatic deposition mask for ten (individually analyzed) noise realizations. The shaded error bars indicate the standard deviation. The black dashed line indicates the mean scan termination time. (d) The mean contrast‐to‐noise ratio for ten (individually analyzed) noise realizations. The shaded error bars indicate the standard deviation. The black dashed line indicates the mean scan termination time. [Color figure can be viewed at wileyonlinelibrary.com]

An example of a patient activity distribution with a high‐concentration extrahepatic deposition is shown in Fig. 7. Evident is that the extrahepatic deposition barely changes in shape and size over time, which is also illustrated by the graphs on the relative change in the extrahepatic deposition mask. The mean scan termination time for this activity distribution was 5.7 ± 1.1 min. This shows that for a more complex activity distribution (i.e., not spherical depositions as in the simulation and phantom studies), the scan might also have been earlier terminated.

**Fig. 8:**
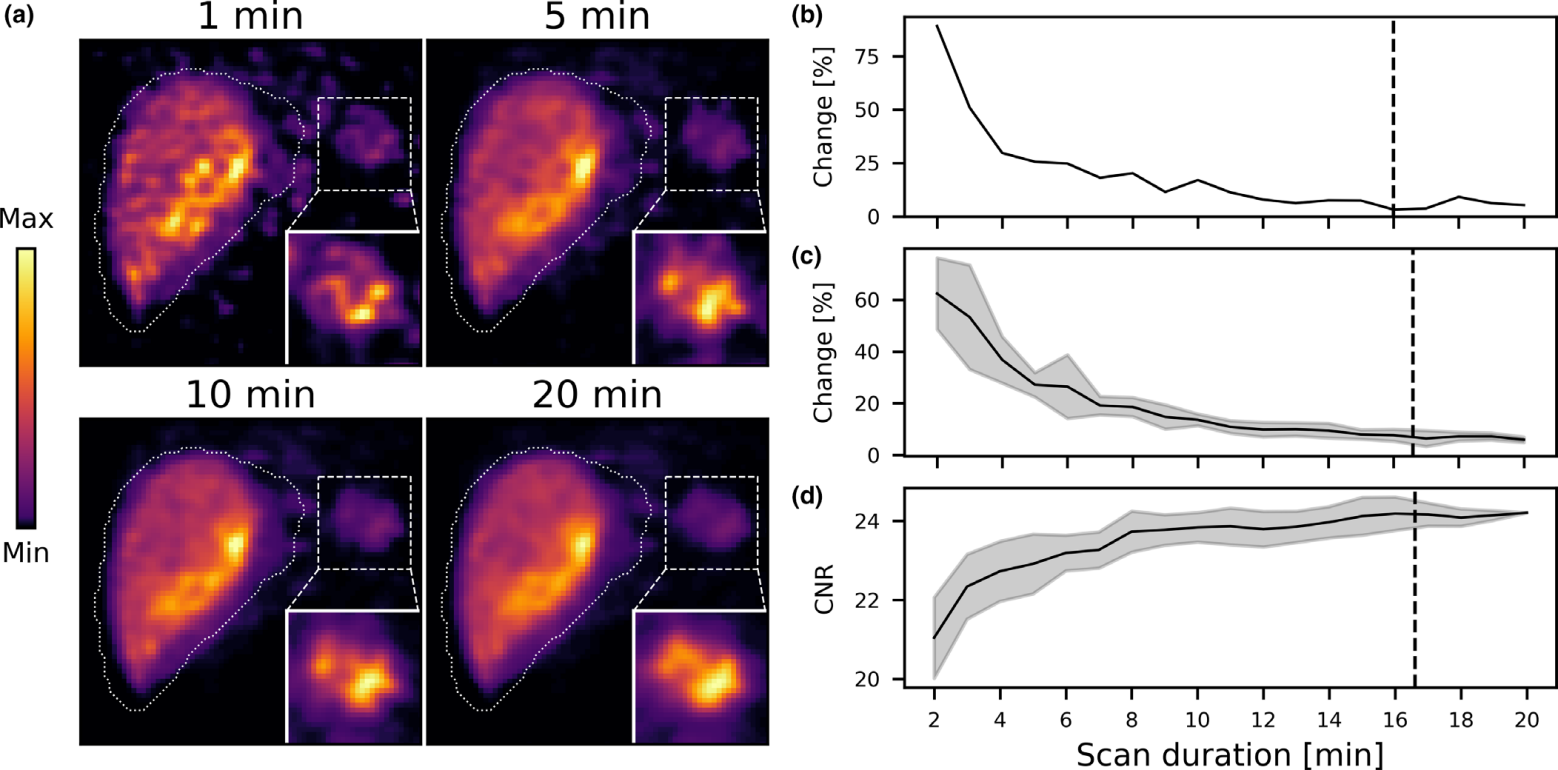
(a) Slices from the reconstructions for four scan durations for a patient activity distribution with a low‐concentration of extrahepatic deposition. (b) The relative change in the mask of the extrahepatic deposition, as a function of scan duration. The black dashed line indicates the scan termination time. (c) The mean relative change in the extrahepatic deposition mask for (individually analyzed) ten noise realizations. The shaded error bars indicate the standard deviation. The black dashed line indicates the mean scan termination time. (d) The mean contrast‐to‐noise ratio for ten (individually analyzed) noise realizations. The shaded error bars indicate the standard deviation. The black dashed line indicates the mean scan termination time. [Color figure can be viewed at wileyonlinelibrary.com]

An example of a patient activity distribution with a low‐concentration of extrahepatic deposition is shown in Fig. 8. For this activity distribution, the shape of the extrahepatic deposition does change considerably over time. The mean scan termination time for this activity distribution was 16.6 ± 3.4 min. This illustrates that large differences in the scan termination times can be expected for patient activity distributions.

**Fig. 9:**
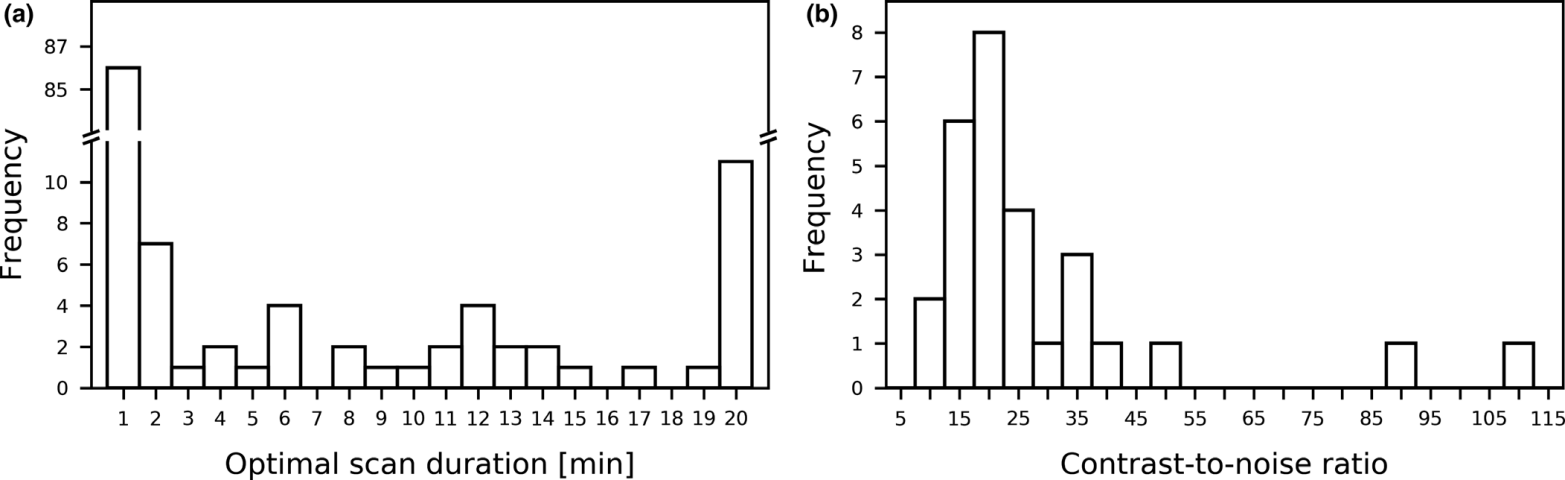
(a) The scan termination times for the 129 patient activity distributions for one noise realization. (b) The contrast‐to‐noise ratios of the extrahepatic depositions at the scan termination times for one noise realization.

The collection of scan termination times for all patient scans (for a single noise realization) is shown in Fig. 9(a). A large fraction of the activity distributions was already terminated at 1 min; these are the activity distributions that had < 0.075% total extrahepatic activity after one rotation. A substantial fraction of the activity distributions did not converge at 20 min; these scans might have benefited from a longer scan duration.

For the 129 activity distributions, 28 extrahepatic depositions were detected in 25 patients for the 20‐min scans. Adaptive scanning accurately detected all depositions in all noise realizations (using the 20‐min scans as the ground truth). The CNRs of the detected extrahepatic depositions at the scan termination times are shown (for a single noise realization) in Fig. 9(b). All extrahepatic depositions in all noise realizations had a CNR > 4 and hence should also be visually detectable.^9^

The mean scan durations and the mask and activity differences of the extrahepatic depositions at the scan termination times with the 20‐min scan are shown in Table IV for the individual noise realizations. The mean scan duration was 4.8 ± 0.2 min, the mean difference in the mask with the full‐duration scan was 7.0 ± 1.0%, and the mean activity difference was 2.0 ± 0.5%.

**Table 4:** For adaptive scanning, shown are the mean scan duration and the difference in the extrahepatic deposition mask and the extrahepatic activity with the 20‐min scan. For scanning with a single‐rotation, shown is the minimum scan duration required for accurate detection of all extrahepatic depositions.

Noise realization	Adaptive scanning			Single‐rotation
	Mean scan duration (min)	Mean mask difference (%)	Mean activity difference (%)	Minimum required scan duration (min)
1	4.7	5.8	1.9	20
2	4.4	8.7	2.7	20
3	4.8	6.2	2.0	18
4	5.1	5.7	2.2	18
5	4.8	6.8	1.1	18
6	4.9	7.8	2.0	20
7	5.0	6.5	1.5	20
8	4.8	7.9	2.7	17
9	4.8	6.3	1.3	19
10	4.8	8.0	2.4	18
Mean	4.8 ± 0.2	7.0 ± 1.0	2.0 ± 0.5	18.8 ± 1.1

The minimum scan durations (with a single‐rotation) required for the accurate detection of all extrahepatic depositions (using the same detection criteria as with adaptive scanning) are also shown in Table IV. Several noise realizations could not be shortened, that is, they require the full 20‐min scan for accurate detection. In the remaining noise realizations, a scan shortening of only a few minutes could be achieved. The mean minimum scan duration required for scanning with a single‐rotation was 18.8 ± 1.1 min.

In conclusion, the switch from a single‐rotation scan to an adaptive scanning protocol allows the mean scan duration (for the studied patient distributions) to be reduced from 18.8 ± 1.1 min to 4.8 ± 0.2 min, without compromising the detectability of extrahepatic depositions.

## 4 Discussion

We investigated the potential for an adaptive scan duration protocol in SPECT for the radioembolization pretreatment procedure and showed the feasibility of terminating scans at an earlier point in time without affecting the detectability of extrahepatic depositions.

We believe that our approach is not limited to the detection of extrahepatic depositions. As long as a measure of interest is available, adaptive scanning can be performed to shorten the scan duration. For instance, we envision that it could be used in cardiac SPECT since quantitative measures are often evaluated in this protocol. However, we acknowledge that some protocols do not have clearly defined objectives and adaptive scanning would not be possible for these cases.

We also acknowledge that the scan duration range that can be achieved in clinical practice is limited. The workup of a patient (positioning, providing instructions, etc.) requires several minutes and would become the limiting factor for very fast (1‐min) scans. Moreover, patients can only lie still for a certain amount of time (usually approximately 30 min) and will start moving for longer scans. It may be beneficial to set a minimum and maximum scan duration to ensure that such practical considerations do not interfere with the scanning workflow.

Changes would need to be made to the patient and technician scheduling if adaptive scanning is employed in clinical practice since the scan start and finish times are no longer known in advance. Rather than scanning a patient on a specific scanner at a specific time, the patient would be scanned in a certain timeslot on whichever scanner is first available. This form of scheduling becomes increasingly practical when more scanners are available since scan duration extremities would have less impact on the total workflow.

A specific case that does not require scheduling changes would be the use of adaptive scanning in interventional SPECT. Our group is developing a mobile, compact SPECT scanner that can be used in the intervention room.^10^, ^11^, ^12^ We envision that this system could be used to perform a SPECT scan after every ^99m^Tc‐MAA injection in the pretreatment procedure of radioembolization so that the response to every injection is individually monitored. For this purpose, adaptive scanning would be beneficial in minimizing the total time spent in the intervention room.

The method in this work has been developed only for the detection of extrahepatic depositions since this is the most common objective for the pretreatment procedure of radioembolization. There are, however, also institutes where the ^99m^Tc‐MAA scan is used to assess the perfusion of target lesions (with potential necrotic cores) and the activity to the parenchyma. Furthermore, the potential for the ^99m^Tc‐MAA scan to be used for dosimetry planning is being investigated within trials.^13^, ^14^ Our algorithm does not take such (intrahepatic) measures into account and hence should be modified if the above methods are introduced in regular clinical practice.

A pre‐requisite for adaptive scanning to function is that the reconstructions are performed in a fast manner. The current clinical reconstruction software takes approximately a minute to finish, which is likely sufficient for use in clinical practice. Further speed‐up may, for instance, be achieved by employing GPU‐based software^15^ or deep learning approaches.^16^

A further requirement for adaptive scanning to be used in the pretreatment procedure of radioembolization is that the liver and lung delineations need to be present in advance. The lung delineations can be normally be created quickly via thresholding of the Hounsfield units of the low‐dose CT. For liver delineation, atlas‐based methods are often employed. Since the liver and lung delineations are dilated by the relatively large margin of 2 cm to cope with partial volume effects and respiratory motion, small dilatation errors are not expected to substantially alter the results.

It was shown that fast scanner rotation resulted in a slightly decreased reconstruction resolution. In clinical practice, however, patient motion is expected to be the major limiting factor for resolution and it is hence not expected that severe problems would arise from the fast detector rotation. If it does prove that the fast detector rotation is undesired, the rotation speed could simply be lowered somewhat (e.g., making rotations of 2 min each).

The threshold value for the relative change in the mask to consider the scan converged is linked to the objective that one wants to achieve. A lower threshold provides reconstructions with a higher confidence on the imaging task, a higher threshold allows for faster scans. We chose the 5% threshold in this work because with this value all extrahepatic depositions in the patient scans were accurately detected. Eventually, it depends on the preference of the physician which extrahepatic depositions (e.g., which activity and volume) need to be detectable and which ones can be safely ignored.

Other methods for adaptive scanning have been previously developed (primarily in positron‐emission tomography).^17^, ^18^ The majority of these methods are projection‐based, that is, the number of photons collected is measured in the first few projections in a certain region of interest, from which the optimal scan duration is then derived. We cannot use these methods for two reasons. First, it is unknown in advance where an extrahepatic deposition will arise and hence it is not possible to focus on a certain region of interest beforehand. Second, due to the limited intrinsic spatial resolution and the substantial scatter contribution of the SPECT projections, it is challenging to detect small activity depositions. For these reasons, we believe that the reconstruction‐based method as described in this work will outperform projection‐based ones.

The relationships between the extrahepatic deposition characteristics (size, activity concentration, location) and resulting optimal scan duration were only studied in the simulation study since the sample size of the patient distributions was too low to obtain statistically significant results. It is interesting to revisit the relationships when more patient data becomes available.

The optimal scan durations obtained in the patient study showed that for a substantial fraction of the distributions, the scans could have been terminated after 1 min without affecting the extrahepatic detection accuracy. The study of Van der Velden *et al.* ^2^ showed that the LSF estimation has an error of less than 0.5% point with the ground truth of 5% for a 1 min scan. The LSF estimation is hence expected to be sufficiently accurate for use in clinical practice, even for the very short scans as studied in this work.

In our institute, only one SPECT scan is made per radioembolization procedure since the field of view of the detectors is sufficiently large to cover all potential extrahepatic deposition origins. If one, however, wants to visualize multiple body regions (e.g., because of a smaller detector coverage or a large patient), more acquisitions can be made. These scans can be performed with the same methodology as described in this work and should be separately analyzed.

The approach in this work investigated a scanner with a rotating detector because such scanners are most used in current clinical practice. SPECT scanners with static detectors are, however, becoming increasingly available (e.g., the D‐SPECT for cardiac imaging or the VERITON for full‐body imaging; both developed by Spectrum Dynamics Medical). Such devices could similarly benefit from adaptive scanning by evaluating the list‐mode acquisition, for example, every minute and are furthermore not constrained by the scanner mechanics.

## 5 Conclusions

The feasibility of an adaptive SPECT scan duration (by assessing the activity distribution characteristics during scanning) was demonstrated for the detection of extrahepatic depositions in the pretreatment procedure of radioembolization. We believe that our approach could also benefit other forms of SPECT scanning, as long as a measure of interest is available for optimization.

## Conflict of Interests

This project has received funding from the European Union’s Horizon 2020 research and innovation program under grant agreement No 646734. No other potential conflicts of interest relevant to this article exist.
